# Method of Step Detection and Counting Based on Measurements of Magnetic Field Variations

**DOI:** 10.3390/s21237775

**Published:** 2021-11-23

**Authors:** Patryk Łaś, Piotr Wiśniowski

**Affiliations:** Institute of Electronics, AGH University of Science and Technology, Al. Mickiewicza 30, 30-059 Krakow, Poland; las@agh.edu.pl

**Keywords:** magnetic sensor, motion detection, step detection, gait, HAR, activity recognition

## Abstract

Basic human activity recognition (HAR) and analysis is becoming a key aspect of tracking and identifying daily habits that can have a critical impact on healthy lifestyles by providing feedback on health status and warning of deterioration. However, current approaches for detecting basic activities such as movements or steps rely on solutions with multiple sensors which affect their size and power consumption. In this paper, we propose a novel method that uses only a single magnetic field sensor for basic step detection, unlike the well-known multisensory solutions. The approach presented here is based on real-time analysis of magnetic field sensor measurements to detect and count steps during a walking activity. The approach is implemented in a system that integrates a digital magnetic field sensor with software blocks: filter, steady state detector, extrema detector with classifier, and threshold comparator implemented in an embedded platform. Outdoor experiments with volunteers of different ages and genders walking at variable speeds showed that the proposed detection method achieves up to 98% accuracy in step detection. The obtained results show that a single magnetic field sensor can be used to detect steps, and in general offers the possibility of simplifying the current solutions by reducing the device dimensions, the cost of a system and its power consumption.

## 1. Introduction

In recent years, human activity recognition (HAR) [1] has become an important topic of scientific research due to the growing interest in healthy lifestyles associated with physical activity. The ability to detect a lack of, or minimal, physical activity has become a key factor in the care of the elderly or people after hospitalization, for whom irregularity or decrease in daily activity is the first symptom of deteriorating health. In addition, expanding technological progress has led to an increase in the number of services provided through computers, limiting our daily activity habits and causing the number of people suffering from diseases of the vascular system to increase year by year. These health-related reasons have led to the development of a number of different methods of activity detection based on a wide range of sensors [2]. From a large group of integrated, widely available sensors, the most common approach of HAR is the utilization of multiaxis inertial sensors [3,4,5] combined with advanced classification techniques such as Bayesian decision making (BDM), least-squares method (LSM), k-nearest neighbor algorithm (k-NN), dynamic time warping (DTW), support vector machines (SVM) and artificial neural networks (ANN), which have high computation costs but can reach up to 99.6% detection accuracy.

One of the most basic and crucial aspects related to physical activity recognition and preliminary classification is motion detection. In the basic HAR solution, motion is treated as a displacement, which at a fundamental level requires the detection and counting of steps in a given period of time. Common approaches to step detection use signals from acceleration sensors [6,7,8] where all 3D axis measurements are required at real-time computation level and reach nearly above 90% of detection accuracy. Sometimes a single acceleration sensor approach is extended with additional gyroscopic [9] or magnetic [10] sensing to improve the step detection ratio. However, the use of a standalone magnetic field sensor for HAR has not yet been reported. For example, in [11], the authors presented a direction change detection algorithm based on magnetic field sensor measurements that achieved high detection accuracy after initial calibration. The researchers in [12] used an additional magnet attached to the shoe to generate a magnetic field disturbance that enabled step detection by a magnetic field sensor mounted on the other shoe and allowed the traveled distance to be calculated. In the experiment, they achieved an accuracy of 0.3 m walking along a loop totaling 66 m in length. Meanwhile, in [13], the authors present a complex system for basic electronic devices and activity recognition based on a group of magnetic field sensors located on different parts of the human hand. Despite the various applications of magnetic field sensors in HAR, the direct step detection and counting based on magnetic field variations has not yet been directly addressed.

However, the research trends in the improvement and development of magnetic field sensors have increased their resolution and minimal detection threshold [14]. Thus, they could be a good replacement for current solutions in certain areas of activity detection. Therefore, we present an approach for step detection and counting based on real-time processing and analysis of magnetic field variation measured by magnetic field sensor. This approach was implemented using a well-known and widely used embedded platform and a commercial digital magnetic field sensor. By combining a magnetic field sensor with the proposed complex multiblock signal processing algorithm, high step detection accuracy (98%) was achieved. The obtained results show that a single magnetic field sensor can be used to detect steps, and in general, this offers the possibility of simplifying the current solutions. Moreover, the block design of the proposed approach allows the different processing parts to be easily replaced, which can significantly reduce the processing time or the power consumption of the whole system in certain applications without compromising the accuracy of step detection, which is a key factor in systems with stringent and limited power and energy requirements.

## 2. Methods

**Figure 1:**
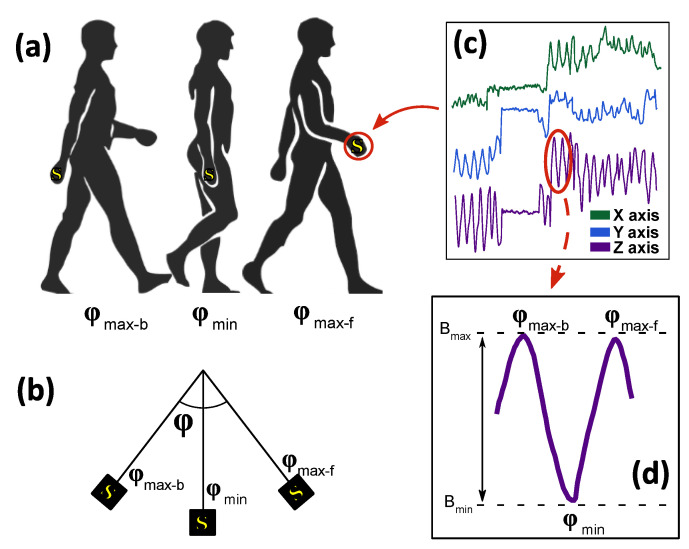
Concept of step detection based on measurement of magnetic field variation caused by natural arm swing during walking. Position of sensor on the arm (**a**). The arm as a pendulum-like motion (**b**). Example of magnetic field measured by the sensor during walking (**c**). Periodically changing magnetic field caused by the arm swing during walking (**d**).

We used the natural arm movement during walking in our magnetic field sensor-based approach to step detection and counting (Figure 1). The arm oscillates out of phase during walking, i.e., the left arm moves forward when the right leg and torso move forward and vice versa for the opposite leg and arm [15]. This arm motion can be considered pendulum-like [16,17] with maximum forward (max-f) and backward (max-b) positions. To use the arm swing for step detection, we attached a magnetic field sensor (S) to the hand (wrist position) (Figure 1a). The movement of the sensor between max-f and max-b in the Earth’s magnetic field (Figure 1b) causes a change in the components of the magnetic field (Figure 1c). The sensor measures the change in the magnetic field between B_max_ and B_min_ (Figure 1d). B_max_ corresponds to the maximum forward and backward movement of the arm and B_min_ corresponds to the perpendicular position of the arm to the ground. Due to the out-of-phase relationship between arm and leg movement, the measured periodic change in magnetic field can be related to leg movement, i.e., steps.

**Figure 2:**
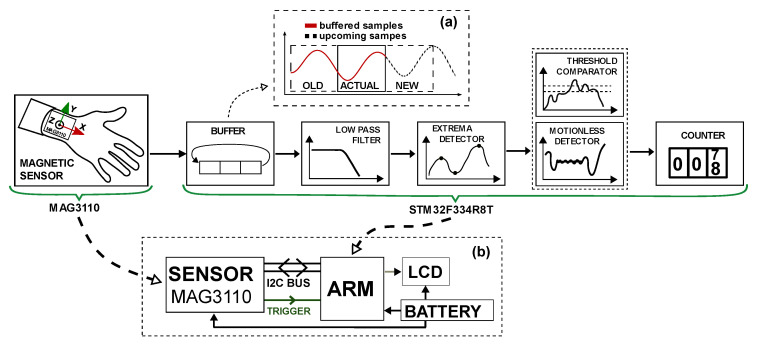
System hardware and functional blocks for data processing implemented in the microcontroller. Circular buffer memory section (**a**). System components: communication and power bus wiring (**b**).

To investigate the possibility of step detection using a magnetic field sensor, we integrated a magnetic field sensor with an embedded platform. We used a 3-axis magnetic field sensor MAG3110 attached to the hand (wrist position). The sensor can measure magnetic field in the range of +/− 1000 uT with a sensitivity of 0.1 uT and has noise level of 0.25 uT [18]. It was configured to acquire data in full range at a sampling frequency of 40 Hz and a resolution of 16 bits. The sensor measured the instantaneous magnetic field variations caused by walking (Figure 1c). The recorded field variations show that the Z-axis was most affected by walking, which gave us the basis to analyze only data from that axis. We used the STM32F334R8T (ARM Cortex M4F) microcontroller [19] as the main processing unit. The microcontroller gathers magnetic field measurements collected by sensor over digital I^2^C communication bus (Figure 2b). When measurement records are ready to read, the sensor triggers a data reception process toggling the interrupt line connected to the microcontroller. This additional connection provides synchronization between sensor and controller and ensures that measurement record will not be missed or readout twice. The recorded magnetic field variations are processed in several functional blocks (Figure 2) designed for collecting magnetic field samples, filtering the collected data, and associating the magnetic field variations with the steps covered during the walking period.

The block circular buffer enabled us to preserve historical data while collecting new, upcoming samples without impacting on currently processed data. We implemented it as a circular buffer. The proposed implementation divides the main circular buffer space into three subblocks (Figure 2a). Each of these blocks stores data as follows: historical data (already processed), current data (ready for processing), and new pending data (collected for future processing). This approach allows our system to store historical data for a short period of time, which becomes part of the feedback loop for the following functional block of the algorithm. In the block circular buffer approach, we can distinguish two phases of data aggregation. The first phase represents the period of actual data processing and collection of new data. The second phase, the buffer switching phase, represents the action of overwriting blocks, where the current data that have already been processed replace the historical data. New data that have just been collected become the record to be processed, while the content of the historical block is cleared and prepared for the collection of new datasets.

A windowing FIR filter reduced the output signal edge effect and decreased the number of operations by removing higher frequency components from the magnetic field signal. It combines a regular FIR filter approach with data windowing. This method consists of two filtering phases—first, the historical data are filtered to prepare the start-up condition of the filter, and then the actual data are processed. We chose this solution to reduce the edge effect of data filtering caused by sample-less starting conditions. Although the windowing of an operational buffer is twice as large, it can significantly reduce the number of filtering operations. This reduction results from the block filtering instead of continuous filtering, which would be triggered each time a new sample is received. In addition, the windowing can be directly connected to a circular block buffer, which reduces the number of operations required to prepare the data for filtering. What is more, filtering removes higher frequency components from the signal, which can significantly reduce the complexity of step detection in the next processing block.

**Figure 3:**
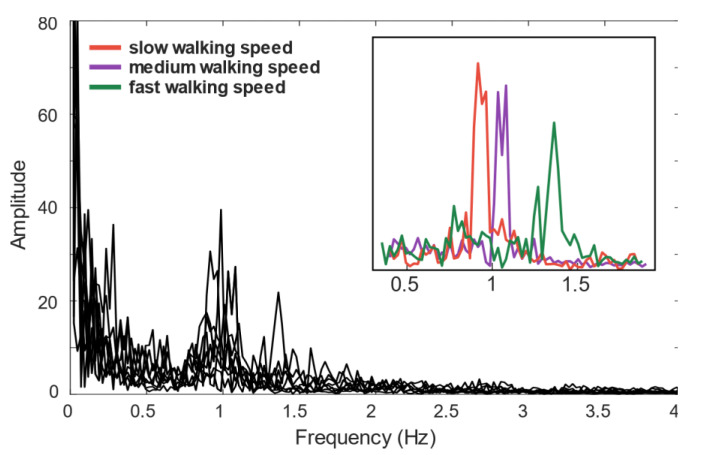
Influence of walking speed on FFT spectra of magnetic field variation during walking activity.

FFT analysis showed that the cutoff frequency of the filter set to 2 Hz removes higher frequency components without losing step detection information. The spectrum of recordings from several walking sample records (Figure 3) shows that walking at different speeds significantly affects the frequency components below 2 Hz (Figure 3-inset). The walking speed in relation to frequency can be calculated by the following equation:

(1) $$\mathrm{frequency}\left[\mathrm{Hz}\right]=\frac{\mathrm{walk}\mathrm{speed}\left[\frac{m}{s}\right]}{\mathrm{stride}\mathrm{length}\left[m\right]}$$

Assuming that the average walking speed varied in the range 4–6.5 km/h and average stride length is 1.3 m [20], the frequencies correlated with walking are in the range of 0.85–1.38 Hz. For this frequency, we designed an FIR filter with a cutoff of 2 Hz. The best filtering results were obtained with a filter order above 45. Accordingly, we chose a filter order of 47, which provides the best balance between filtering performance and filter memory requirements.

The extrema detector identified all peaks and valleys in the buffered data. All points detected in the filtered low frequency signal points are first classified as potential steps. We implemented a detection algorithm with a simple amplitude comparator that compares the amplitude of the point with the values of its surrounding neighbors. If the neighboring values are lower (for peaks) or higher (for valleys), the point under investigation is classified as a local extremum.

The threshold comparator enabled the first level extrema to be filtered. We apply two threshold classifiers that compare each detected extremum with the following one. The first compares the amplitudes between two adjacent extrema provided by the second classifier. If the differences between the tested extrema are smaller than the required threshold, the next complementary extremum is included in a comparison test. The second classificator is responsible for providing a complementary extremum for the threshold test. In this test, the complementary extremum should be understood as the opposite type to the currently tested sample—peak for valley and valley for peak. Such an approach reduces the number of tested extrema, since all adjacent signal extrema are of the opposite type (repeating the peak–valley–peak–valley pattern). In this case, the rejection of a sample forces the algorithm to skip the following sample, which is always of the same type. This procedure significantly reduces the number of possible steps and eliminates false steps caused by variations in the ambient magnetic field.

The motionless detector allowed a final step classification. Observed low amplitude variations during step recognition can be classified as motionless phase operation. Distinguishing whether the currently processed dataset corresponds to a movement or motionless action allows us to exclude some extrema from the final processing stage. In the present case, the motionless phase can be directly assigned to the walk halt. Thanks to this observation, we could apply a simple absolute threshold comparator to discard extrema representing false steps during standing. The absolute threshold was calculated as the average sample deviation from different sets of samples collected during the motionless phase and used as a constant comparison value.

## 3. Results

**Figure 4:**
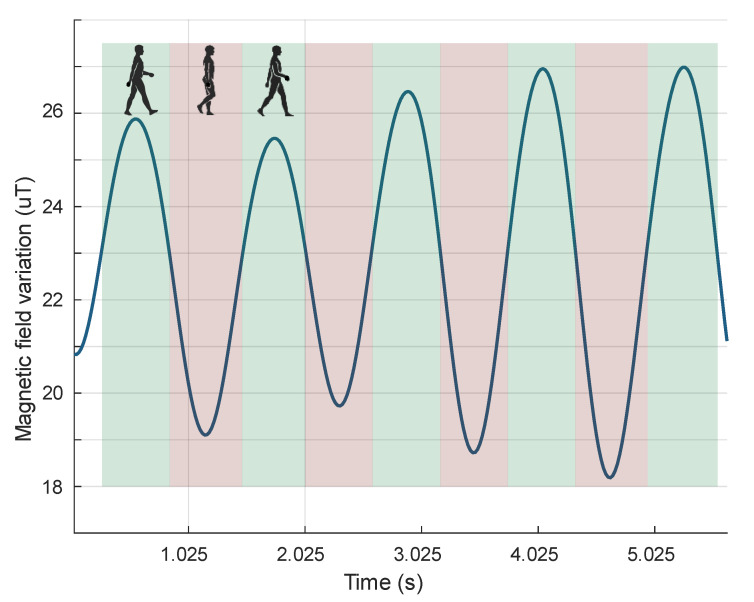
Magnetic field signal variations after low pass filtering. The signal extremum correlates with leg movement during steps.

Low frequency magnetic field variations correlate with steps taken during walking (Figure 4). Magnetic field samples recorded by a wrist-mounted sensor—with the Z-axis oriented perpendicular to the direction of movement and parallel to the ground surface—show significant field variations during walking. The records with filtered out neighbor signal extrema that differ by less than 5 uT show the link between steps and signal changes. Analysis of these changes revealed a direct correlation between the local extrema of the signal and the steps taken.

**Figure 5:**
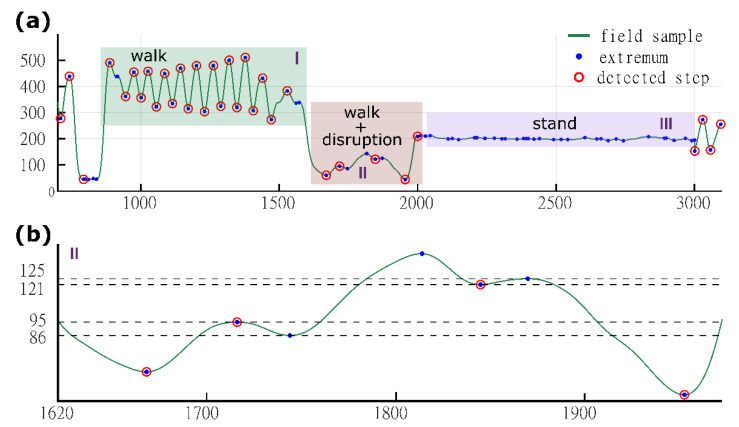
Extrema filtration and step detection in the low frequency component of magnetic field signal variation recorded during complex walk activity (**a**). Threshold discriminator for fake extrema rejection (**b**).

Extrema in magnetic field variations caused by walking can be related to the steps taken. Long term (approx. 1 min) recording of filtered magnetic field samples (Figure 5a) shows a deviation in relative extrema levels during mixed walking and standing action. Analysis of these changes led to the need to distinguish three different approaches to step detection based on extrema. The first is direct extrema mapping, where the detected extrema represent steps. The second, which is more complex, is where additional environmental disturbances must be filtered out before assigning the extrema to steps. In the third method, motionless phases (while standing) are skipped, and the extrema cannot be correlated with steps.

The direct extrema mapping approach allows all detected extrema to be linked to steps during the walking period. This is the simplest method for detecting steps. The direct mapping approach is used when the amplitude difference between two consecutive extrema exceeds 0.5 uT (10 sampling units). The noise of the sensor does not exceed 0.25 uT [18]. The level of fake step (0.5 uT) was determined as the average value of the amplitude difference between two adjacent extrema in multiple test recordings where no ambient (generated by the desired motion) disturbance was detected.

Magnetic field variations disturbed by the ambient field require additional threshold verification to avoid fake step counting. In cases where the signal, even after filtering, contains disturbances that are labelled as extrema (Figure 5b), more complex operations are required to remove additional noisy extrema. Avoiding fake step hits requires additional comparison and shift operations to effectively remove false extrema from the step counting algorithm. Firstly, the difference between adjacent extrema values is compared to check whether the values exceed the fake step threshold (0.5 uT). If the threshold exceeds the comparison value, both extrema are directly mapped to steps. Otherwise, the second extremum is skipped and the next complementary (with the opposite sign of the first derivative) extremum is included in the comparison test. The detection phase is repeated until the complementary extremum passes the threshold test.

A simple step count detection algorithm applied to samples recorded by one axis of the magnetic field sensor can achieve up to 98% accuracy in step detection. The accuracy of the detection was demonstrated by experiments (Table 1). To obtain reliable results, experiments were conducted with a group of seven volunteers of different ages (21–82) and genders walking at different speeds. The average walk speed during the test was measured by a smartphone application utilizing GPS measurements. Each volunteer was asked to perform three trials in each of two typical environments—except 2 performed by elderly. The first typical environment was a wild nature area where external magnetic field disturbances were minimal. The second was an urban area, where much more artificial disturbances were detected compared with the first location. In each trial, participants were asked to follow any route with his regular walking speed with an experimental step detection system on their hand (wrist area). Participants did not show any symptoms of disorder manifested by unconditional or involuntary hand movements. During the test, any kind of gesticulation or abnormal hand movement was forbidden and the subject was required to count the steps performed during the walk.

## 4. Conclusions

This paper proposes a new approach utilizing a single magnetic field sensor for step detection. The approach fills the gap in simple and basic activity detection based on magnetic field variation analysis. Unlike most existing studies and a known solution based on an inertial sensor or a group of sensors (acceleration, gyroscopic or pressure), the proposed approach requires only a single-axis magnetic field sensor and a low power embedded microcontroller.

In the proposed approach, the magnetic field variations caused by walking are continuously measured by a magnetic field sensor and collected in an embedded platform. In the next stage, the detection algorithm is applied on previously buffered data to extract the steps associated with the signal extrema and map them into the performed steps. By using a commonly available embedded platform and a commercial magnetic field sensor, the cost of the proposed step detection system can be significantly reduced. Moreover, the block design of the proposed algorithm allows its different processing parts to be easily replaced, which can significantly reduce the processing time or power consumption of the entire system in specific applications without compromising the accuracy of step detection, which is a key factor in systems with stringent and limited power and energy requirements.

It is also important to indicate that the presented approach based on the analysis of magnetic field variation has certain limitations, despite its high accuracy in step detection. The first limitation is random magnetic field peaks in the environment originating from nearby electronic devices or natural phenomena. These can generate a field fluctuation high enough to induce fake step conditions, which can be treated by the algorithm as true steps recorded during walk. The second limitation is gesticulation or disorders causing involuntary hand shaking. Such a movement performed by a hand equipped with a magnetic field sensor might create a similar magnetic field vector distribution as the body movement during walking and cause a false positive step classification. Another limitation that could be considered in a future work is the method of filtering field variations related to steps performed. The present study focuses on a limited group of healthy individuals with an average stride length (approximately 1.3 m) and a normal walking speed (4.0–6.5 km/h). This results in an additional area of research in the field of filtering method and design for groups of individuals with motor disorders or variable gait patterns that affect step stride length and walking speed.

There are several possible future research directions that can extend the usability of the proposed approach. The first possible direction is to reduce the power consumption, where a digital multi-axis magnetic sensor can be replaced by a single-axis analog sensor. The use of the analog sensor also enables the replacement of the digital filter with its low power hardware substitute. Another possibility is to increase the complexity of activity recognition by extending the detection blocks to detect not only steps but also activity areas (urban, suburban, mountain, …) by applying a more complex harmonic analysis of the magnetic field variations. More complex signal analysis could further increase the immunity of the detection algorithm to random magnetic field fluctuations in the environment, which limit the application area of the presented approach, and improve the accuracy of step detection. Furthermore, such processing can also efficiently mask unwanted variations caused by additional upper limb activity and allow for more complex hand movements, such as gesticulations, without compromising the accuracy of step recognition. Another—and the most advanced—direction that could be considered is the application of machine learning or artificial intelligence algorithms. Such an approach could lead to two different research directions. The first will enable more efficient removal of false extrema representing steps and increase the accuracy of step detection. Secondly it might be possible to detect the first symptoms of a neurological disorder by analyzing abnormal and involuntary hand shaking during walking.

## Figures and Tables

**Table 1:** Worst test results in a set of three trials for all volunteers per each test area.

Gender	Age	Average Speed (km/h)	Test Area ^1,2^	Detected Step Count	Counted Steps	Accuracy (%)
Male	21	5.3	W	216	211	97.7
		5.6	U	155	163	95.1
Male	29	5.4	W	326	332	98.2
		5.1	U	234	251	93.2
Female	43	4.8	W	178	186	95.7
		5.1	U	130	126	96.9
Female	51	5.0	W	287	296	97.0
		4.6	U	236	221	93.6
Male	51	4.5	W	167	175	95.4
		4.6	U	307	294	95.8
Female	82	4.2	W	220	207	93.7
Male	82	4.0	W	81	86	94.2

^1^ W—wild nature area, ^2^ U—urban area.

## Data Availability

Not applicable.
